# Unhealthy white matter connectivity, cognition, and racialization in older adults

**DOI:** 10.1002/alz.13494

**Published:** 2023-10-12

**Authors:** Sarah K. Royse, Beth E. Snitz, James B. Hengenius, Theodore J. Huppert, Rebecca E. Roush, Rebecca E. Ehrenkranz, James D. Wilson, Marnie Bertolet, Alexandria C. Reese, Geraldine Cisneros, Katey Potopenko, James T. Becker, Ann D. Cohen, C. Elizabeth Shaaban

**Affiliations:** ^1^ Department of Epidemiology University of Pittsburgh Pittsburgh Pennsylvania USA; ^2^ Department of Radiology University of Pittsburgh Pittsburgh Pennsylvania USA; ^3^ Department of Neurology University of Pittsburgh Pittsburgh Pennsylvania USA; ^4^ Department of Electrical Engineering University of Pittsburgh Pittsburgh Pennsylvania USA; ^5^ Department of Mathematics and Statistics University of San Francisco San Francisco California USA; ^6^ Department of Biostatistics University of Pittsburgh Pittsburgh Pennsylvania USA; ^7^ Department of Psychology University of Pittsburgh Pittsburgh Pennsylvania USA; ^8^ Department of Psychiatry University of Pittsburgh Pittsburgh Pennsylvania USA

**Keywords:** cerebral small vessel disease, cerebrovascular lesions, cognition, connectome, racialization

## Abstract

**INTRODUCTION:**

White matter hyperintensities (WMH) may promote clinical Alzheimer's disease (AD) disparities between Black American (BA) and non‐Hispanic White (nHW) populations. Using a novel measurement, unhealthy white matter connectivity (UWMC), we interrogated racialized group differences in associations between WMH in AD pathology‐affected regions and cognition.

**METHODS:**

UWMC is the proportion of white matter fibers that pass through WMH for every pair of brain regions. Individual regression models tested associations of UWMC in beta‐amyloid (Aβ) or tau pathology‐affected regions with cognition overall, stratified by racialized group, and with a racialized group interaction.

**RESULTS:**

In 201 older adults ranging from cognitively unimpaired to AD, BA participants exhibited greater UWMC and worse cognition than nHW participants. UWMC was negatively associated with cognition in 17 and 5 Aβ‐ and tau‐affected regions, respectively. Racialization did not modify these relationships.

**DISCUSSION:**

Differential UWMC burden, not differential UWMC‐and‐cognition associations, may drive clinical AD disparities between racialized groups.

**Highlights:**

- Unhealthy white matter connectivity (UWMC) in Alzheimer's disease (AD) pathology–affected brain regions is associated with cognition.
- Relationships between UWMC and cognition are similar between Black American (BA) and non‐Hispanic White (nHW) individuals.
- More UWMC may partially drive higher clinical AD burden in BA versus nHW populations.
- UWMC risk factors, particularly social and environmental, should be identified.

## 1 BACKGROUND

Black American (BA) individuals are two times more likely to have and to develop clinical Alzheimer's disease (AD) compared to those who are non‐Hispanic white (nHW).^1^, ^2^ It is currently unclear why BA populations are disproportionately affected by clinical AD, but one potential driver is white matter hyperintensities (WMH). WMH are markers of cerebral small vessel disease that are common in older adults and associated with cognitive decline.^3^ Notably, WMH burden is more severe in BA individuals than in those who are nHW^4^, ^5^, ^6^ and may confer comparatively greater risk of cognitive impairments.^7^, ^8^

In both BA and nHW populations, there is growing interest in the overlap between WMH and clinical and pathological AD as they share many of the same risk factors.^9^ WMH and AD pathology also often regionally co‐occur.^10^ Mechanistically, there are at least two ways in which WMH may influence clinical and pathological AD. First, because of their potential influence on white matter, WMH may impede neuronal communication within and between brain regions, leading to loss of neuronal function and eventual cognitive decline.^11^ Second, and perhaps in addition to independent effects of WMH on cognition, WMH and AD pathology may each influence the progression of the other. For example, it is possible that extracellular beta‐amyloid (Aβ) plaques accelerate WMH by inducing ischemia.^12^ In turn, WMH may promote Aβ or hyperphosphorylated tau aggregation by reducing Aβ clearance or inducing white matter injury, respectively.^13^, ^14^

Previous work has been integral in identifying associations of WMH with pathological and clinical AD, but is limited by the use of crude measurements of WMH burden. That is, most studies have examined these relationships using global or lobar WMH volumes. More spatially precise measurements of WMH would allow for better understanding of the neurobiology underlying WMH in clinical and pathological AD among both BA and nHW populations. For this reason, we leveraged multimodal neuroimaging methods to develop a novel measurement for quantifying WMH burden on specific white matter fibers: unhealthy white matter connectivity (UWMC). This new method was motivated by the hypothesis that WMHs disrupt intercellular connection and communication, which leads to eventual loss of neuronal function.^11^, ^15^ As such, we consider white matter tracts that pass through WMH “unhealthy,” whereas those that do not pass through WMH are “healthy.” For a pair of brain regions, UWMC is defined as the proportion of total white matter fibers that pass through WMH.

Herein, we applied our novel UWMC method to interrogate the role of WMH on fibers connecting AD pathology–affected brain regions in clinical AD disparities. Consistent with previous evidence of independent relationships of WMH with Aβ^12^ and tau,^13^, ^14^ we quantified UWMC in Aβ‐ and tau‐affected regions separately and tested (1) associations of UWMC in AD pathology‐affected regions with cognition and (2) differences in UWMC‐and‐cognition associations between BA and nHW participants.

## 2 METHODS

### 2.1 Study population

For this cross‐sectional analysis, we used data collected in the Connectomics of Brain Aging (CoBRA) project, an ongoing longitudinal study of brain structure and function in community‐dwelling older adults.^16^ Beginning in 2017, participants were recruited from the Pittsburgh, Pennsylvania area via word of mouth, the University of Pittsburgh Alzheimer's Disease Research Center (ADRC), the community‐based Pitt + Me research recruitment web portal, or other ongoing cohort studies of aging, including the Long Life Family Study,^17^ and the Heart Strategies Concentrating on Risk Evaluation (SCORE) study.^18^ Individuals aged 50 to 89 years were eligible for study participation; those with contraindications for magnetic resonance imaging (MRI), history of stroke, or presence of significant psychiatric, neurologic, or unstable medical conditions which affect neuropsychologic assessment or brain structure or function were excluded.

### 2.2 Image acquisition

Each participant acquired an MRI at the Magnetic Resonance Research Center (MRRC) at University of Pittsburgh on a 3T PRISMA scanner (Siemens AG) using a Siemens 64‐channel head coil. Standard sequences from the Human Connectome Project, Lifespan protocol were collected, including structural T1 magnetization‐prepared rapid gradient echo (MPRAGE) images (repetition time [TR] = 2400 ms; echo time [TE] = 2.22 ms; field of view [FOV] = 256 × 256 mm; voxel size = 0.8 mm × 0.8 mm; slice thickness = 0.8 mm) and T2 fluid‐attenuated inversion recovery (FLAIR) images (TR = 9690 ms; TE = 91 msec; FOV = 256 × 256 mm; voxel size = 0.8 mm × 0.8 mm; slice thickness = 0.8 mm). We also collected diffusion‐weighted images (DWI; TR = 3230 ms; TE = 89.2 ms; FOV = 210 × 210 mm; voxel size = 1.5 mm × 1.5 mm; slice thickness = 1.5 mm).

The MRRC has quality control and quality assurance procedures and American College of Radiology certification in place for all scanners. These include daily signal stability scans for echo planar imaging (1% maximum root mean square over a continuous 30‐minute acquisition with a 64 × 64 matrix size) and daily signal‐to‐noise measurements with the standard radiofrequency head coil. In addition to the daily quality control testing of the MRI scanner, each imaging protocol is examined visually prior to submitting it to the local data archive. The scans are checked immediately by a member of the Imaging Team and repeated if necessary.

In a subset of participants, we quantified Aβ burden using [^11^C]Pittsburgh compound B (PiB) positron emission tomography (PET) imaging.^19^ Methods for image acquisition have been described previously.^20^

### 2.3 Image analysis

We normalized all T1 MPRAGE MRIs to Montreal Neurological Institute (MNI) space using FSL version 6.0.^21^ Regions of interest (ROIs) were defined by processing T1 MPRAGEs through the FreeSurfer version 6.0 pipeline and Desikan–Killiany atlas.^22^ To better demarcate functional subdivisions of the striatum, we replaced FreeSurfer‐derived striatal regions with those of the Imperial College London Clinical Imaging Centre (CIC) Atlas.^23^ For quality control, all parcellations were visually inspected prior to further analysis.

We quantified WMH using an automated in‐house segmentation pipeline that has been published previously by our group.^24^ Briefly, we co‐registered native space T2 FLAIR MRIs to respective native space T1 MPRAGE MRIs using a rigid body registration with a mutual information cost function in FSL version 6.0.^21^, ^25^ We then applied T1 MPRAGE‐to‐MNI transformations to each T2 FLAIR to bring these images into MNI space. Next, we normalized T2 FLAIR images to the mean and standard deviation of voxels segmented by FreeSurfer as cerebellar white matter. We then implemented a fuzzy c‐mean classifier method (MATLAB R2014; The MathWorks Inc.) to assign each cerebral voxel a group membership probability of being high or low intensity; we considered a voxel to be WMH if its probability of being high intensity was > 0.75, where this threshold was chosen by visual assessment. All WMH segmentation maps were visually inspected for quality assurance; we did not perform any manual editing.

[^11^C]PiB PET analyses are described in detail elsewhere.^20^ Briefly, we derived a global index of tracer retention by calculating a volume‐weighted average of standardized uptake value ratios (SUVR) in nine composite regions (anterior cingulate, anterior ventral striatum, superior frontal, orbitofrontal, insula, lateral temporal, parietal, posterior cingulate, precuneus) defined by FreeSurfer and the CIC atlas, normalized to cerebellar gray matter;^20^ individual regions which comprise composite regions (and thus, the global region) are provided in Table S1 in supporting information.

**RESEARCH IN CONTEXT**

1. **Systematic review**: We conducted a literature review using traditional sources (e.g., PubMed). While Alzheimer's disease (AD) disparities may be driven by more severe white matter hyperintensities (WMH) in Black American (BA) versus non‐Hispanic White (nHW) populations, previous studies, which are cited, have examined their role using crude measurements.
2. **Interpretation**: Using our novel unhealthy white matter connectivity (UWMC) measurement, our results suggest that the associations of WMH in beta‐amyloid (Aβ)‐ and tau‐affected brain regions with cognition do not differ by racialized group. Rather, more UWMC in these regions may partially explain higher AD incidence and prevalence in BA versus nHW populations.
3. **Future directions**: In overall and racialized group‐stratified analyses, our findings warrant future cross‐sectional and longitudinal studies that examine (1) UWMC in relation to Aβ and tau deposition and (2) interactions of UWMC with Aβ and tau burden on cognition.

### 2.4 Predictors

#### 2.4.1 Unhealthy white matter connectivity

**FIGURE 1:**
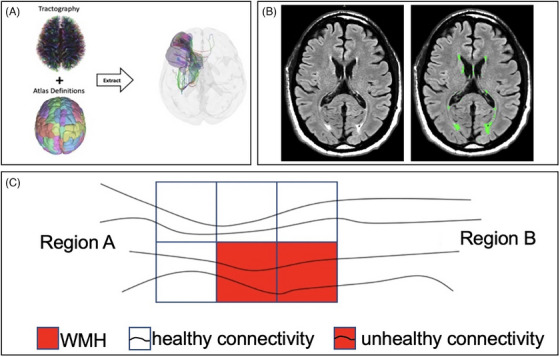
Unhealthy white matter connectivity derivation. A, Pairwise connectivity definitions. B, White matter hyperintensities in a CoBRA participant. C, Example of two connected regions where UWMC = 0.5 (two fibers passing through WMH out of four fibers total). CoBRA, Connectomics of Brain Aging; UWMC, unhealthy white matter connectivity; WMH, white matter hyperintensities

UWMC is a novel method developed by our group. To derive it, we first corrected DWIs for distortion and eddy current using FSL version 6.0.^21^ We quantified white matter connectivity using tractography analyses.^26^ This included performing the following steps on each DWI in DSI Studio: (1) orientation distribution functions (ODFs) were fit to native DWI space, (2) ODFs were transformed into MNI space, and (3) a deterministic streamline propagation technique with a whole‐brain seed generated a full brain connectome;^27^ all connectomes were created in MNI space.^27^ For quality control, we implemented procedures that are built into DSI Studio, including an automated assessment that iteratively tests the goodness‐of‐alignment between the native space DWI and the MNI template and a topology‐informed pruning step that removes artifactual streamlines.^28^ We additionally visually assessed each connectome for artifacts. Resultant streamlines were filtered and classified as either passing or not passing through WMH. We then applied each participant's FreeSurfer and CIC atlas‐derived ROIs to the respective connectome and quantified end‐to‐end white matter connectivity as the total number of streamlines between each pair of ROIs. Finally, we calculated UWMC as the proportion of total streamlines between each pair of ROIs that passed through WMH (Figure 1).

Because we were interested in colocalized UWMC and AD pathology, we extracted individual UWMC between regions characteristically affected by Aβ and tau. For Aβ, we used UWMC between FreeSurfer and CIC atlas ROIs that comprise our global [^11^C]PiB region (Table S1);^20^ for tau, we used those that are included in the meta‐temporal ROI, as has been previously described (Table S2 in supporting information).^29^

We also created lobar UWMC measures for each bilateral lobe by dividing total number of streamlines passing through WMH by total streamlines for all pairwise fiber connections for each pair of ROIs within one lobe.

Finally, we calculated global UWMC as the total number of fibers passing through WMH by total fibers for all pairwise connections for every ROI pair within the entire brain.

#### 2.4.2 Lobar white matter hyperintensity volume

We derived measures of lobar WMH volume for proof‐of‐concept of our UWMC approach. We localized WMH by creating white matter masks from white matter labels and segmentation from FreeSurfer and the International Consortium for Brain Mapping (ICBM) 2009a Nonlinear Symmetric lobar atlas.^30^ We then calculated WMH (mm^3^) in bilateral frontal, temporal, parietal, and occipital lobes (Figure S1 in supporting information). Each lobar WMH volume was normalized to intracranial volume (ICV).

### 2.5 Outcome

At their study visits, participants completed the Montreal Cognitive Assessment (MoCA) as a measure of global cognition.^31^ Potential scores on MoCA range from 0 to 30, where higher scores indicate better cognitive function.

### 2.6 Effect modifier

Each participant self‐reported being BA or nHW. In the context of this study, we refer to these categories as “racialized groups” rather than “race groups” to emphasize that race is a social, rather than a biological, construct. Racialization encapsulates the historical process of individuals being assigned to different groups based on real or imagined phenotypes and, as a result, receiving differential societally‐defined resources and opportunities in legal, political, economic, and medical environments.^32^

### 2.7 Other descriptive variables and potential confounders

We collected several variables to describe our study sample. As described previously, cognitive diagnosis (cognitively unimpaired, mild cognitive impairment [MCI], or AD) was assigned via inter‐disciplinary consensus procedures, either in the ADRC^33^ or in the CoBRA study;^16^ notably, the procedures and the acting neuropsychologist (B.E.S.) were the same at both of these studies. Under both protocols, diagnostic procedures included a comprehensive multi‐domain neuropsychological evaluation, including grade‐level reading as a proxy for educational quality,^34^ self‐reported health history, and consideration of subjectively reported cognitive decline. Published consensus criteria were used for MCI^35^ and for AD^36^ diagnosis. While neuropsychological test norms were not adjusted for race/ethnicity, cognitive adjudication was conducted with consideration of reading level and emphasis on subjective report of cognitive decline to minimize false positives for cognitive impairment classifications.

We also calculated [^11^C]PiB global SUVR as described above. Anyone with a global SUVR ≥ 1.3 was considered [^11^C]PiB positive; this cut‐off was determined using a sparse k‐means clustering and resampling method in a cognitively unimpaired participant sample, as described previously.^37^

Potential confounders were selected a priori using directed acyclic graphs, which were informed by biological plausibility consistent with current scientific literature.^38^ At the time of their MRI visit, participants self‐reported lifetime presence of diabetes mellitus, high cholesterol, and hypertension. Number of hours of physical activity per week was also self‐reported via the Community Healthy Activities Model Program for Seniors (CHAMPS) Questionnaire.^39^ Blood samples were drawn and analyzed with TaqMan Assays to determine apolipoprotein E (*APOE*) allele polymorphisms.^40^ We dichotomously classified each participant as either having or not having an ε4 allele.

### 2.8 Statistical analysis

Within each racialized group, we examined participant characteristics by high versus low global UWMC (low = global UWMC < median; high = global UWMC ≥ median) using Mann–Whitney U, chi‐square, or Fisher exact tests as appropriate.^41^ With the same statistical tests, we additionally tested for differences in participant characteristics between racialized groups. Prior to linear modeling, we log‐transformed physical activity, regional WMH volumes, and UWMC to improve the linearity assumption between predictor variables and MoCA score. We changed all 0 values to 0.05 before log transformations.

**FIGURE 2:**
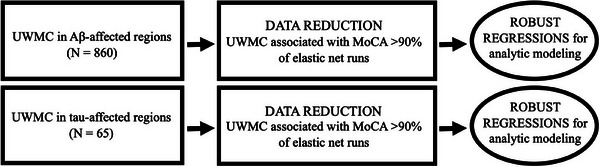
Flow chart of statistical analyses. Aβ, beta‐amyloid; MoCA, Montreal Cognitive Assessment; UWMC, unhealthy white matter connectivity

A flow chart summarizing our analytic approach for testing UWMC‐and‐cognition relationships is provided in Figure 2. Because of the large number of UWMC variables between regions affected by Aβ (*N* = 860) and tau (*N* = 65), we implemented a data reduction step prior to building analytic models.^42^ Our data reduction method was as follows: First, we randomly removed 10% of the data and left the remainder for training. Second, we ran elastic net on the training data set, where the predictors were UWMC in pathology‐specific brain regions and the outcome was MoCA score; the penalty parameter for the elastic net model was chosen by cross‐validation. We repeated steps one and two 120 times and deemed UWMC variables chosen in at least 90% of the elastic net runs as most important predictors of MoCA score and consequently, those that should be included in subsequent individual linear models. We implemented our data reduction method twice: first using UWMC between regions affected by Aβ, then using UWMC between regions affected by tau. For all elastic net runs within the training data sets, we forced main effects of age, sex, racialized group, education, physical activity, diabetes, hypertension, high cholesterol, and *APOE* ε4 into the models.

To test their relationships with MoCA score, we entered the UWMC variables that were selected by the data reduction step into individual robust regression models with the Huber M estimator. We opted to use robust regressions rather than traditional least‐square regression models as these are comparatively more robust against outliers, which we observed in our UWMC data. Because UWMC variables were log transformed prior to model entry, each corresponding beta estimate divided by 100 is interpreted as the difference in MoCA score associated with 1% greater UWMC. We tested each UWMC variable in relation to MoCA score in the full sample and stratified by racialized group. If a relationship was significant in one racialized group, but not the other, we then determined if racialization modified the association between UWMC and MoCA score by testing an UWMC‐by‐racialized group interaction term in the full sample. In all models, we adjusted for age, sex, racialized group, education, physical activity, diabetes, hypertension, high cholesterol, and *APOE* ε4. We controlled for false discovery rate (FDR) using the Benjamini–Hochberg procedure at 5%.^43^

As proof of concept for our UWMC method, we derived Spearman rank‐order correlation statistics to test the degree of similarity between lobar UWMC and WMH volume measurements. We used robust regressions to test the relationship between lobar UWMC and MoCA score in the full sample adjusting for age, sex, racialized group, education, physical activity, diabetes, hypertension, high cholesterol, and *APOE* ε4. We repeated these models stratified by racialized group. If results differed between groups (e.g., if relationship was significant in one racialized group, but not the other), we then tested for an interaction in the full sample. We repeated this approach with lobar WMH volume as the predictor.

We conducted statistical analyses using SAS Software version 9.4 (SAS Institute) and Stata version 17 (StataCorp). For all statistical analyses, we implemented code review procedures.^44^

## 3 RESULTS

At the time of our analysis, 270 participants were enrolled in CoBRA. We excluded participants if they self‐identified as belonging to a racialized group other than BA or nHW (*n* = 6), if they had not yet undergone neuroimaging (*n* = 31), if they had not yet been administered MoCA (*n* = 2), and/or if they had not yet received cognitive diagnosis (*n* = 1). Among those remaining (*n* = 238), we additionally excluded participants with missing data for any confounder variable (*n* = 37). This resulted in a final analytic sample of *n* = 201.

Participant characteristics are presented in Table 1, overall and stratified by racialized group and median global UWMC. In the full cohort, nearly half of the participants self‐reported being BA (49.7%). Participants had a median (Q1, Q3) age of 62.0 years (57.0, 70.0), were predominantly female (66.7%), had a median (Q1, Q3) MoCA score of 25.0 (23.0, 27.0), and were mostly cognitively unimpaired (73.1% vs. 24.9% MCI, 2.0% AD). Among those who underwent PET imaging (*n* = 179), median (Q1, Q3) [^11^C]PiB SUVR was 1.1 (1.1, 1.2), with 16.2% of participants being classified [^11^C]PiB positive.

**TABLE 1:** Characteristics of CoBRA participants at time of neuroimaging visit.

		Black American *N* = 100		Non‐Hispanic White *N* = 101	
	Total *N* = 201	Low global UWMC *N* = 44	High global UWMC *N* = 56	Low global UWMC *N* = 57	High global UWMC *N* = 44
**Demographics**					
Age^*^ (years)	62.0 (57.0, 70.0)	60.0 (55.0, 65.0)	61.0 (56.0, 67.0)	66.0 (60.0, 73.0)	62.5 (56.0, 71.5)
Female^†^	134 (66.7%)	31 (70.5%)	40 (71.4%)	29 (50.9%)^¶^	34 (77.3%)^¶^
Education^*^ (years)	14.0 (12.0, 16.0)	14.0 (12.0, 16.0)	14.0 (12.0, 16.0)	14.0 (12.0, 18.0)^¶^	13.5 (12.0, 16.0)^¶^
Hours of physical activity per week^*^	9.3 (3.8, 18.0)	9.3 (4.6, 16.5)	7.1 (1.8, 12.9)	13.0 (7.3, 20.5)^¶^	9.9 (3.6, 14.6)^¶^
**Cardiovascular risks**					
Hypertension^†^	77 (38.3%)	25 (56.8%)	22 (39.3%)	16 (28.1%)	14 (31.8%)
Diabetes^†^	34 (16.9%)	11 (25.0%)	13 (23.2%)	8 (14.0%)	2 (4.6%)
High cholesterol^†^	71 (35.3%)	15 (34.1%)	19 (33.9%)	22 (38.6%)	15 (34.1%)
**Cognition**					
MoCA score^*^	25.0 (23.0, 27.0)	26.0 (23.0, 27.0)^¶^	23.5 (22.0, 25.0)^¶^	27.0 (25.0, 28.0)^¶^	26.0 (22.0, 27.0)^¶^
Diagnosis^†^					
Alzheimer's disease	4 (2.0%)	1 (2.3%)^¶^	1 (1.8%)^¶^	0 (0%)	2 (4.6%)
MCI	50 (24.9%)	4 (9.1%)^¶^	23 (41.1%)^¶^	13 (22.9%)	10 (22.7%)
Cognitively unimpaired	147 (73.1%)	39 (88.6%)^¶^	32 (57.1%)^¶^	44 (77.2%)	32 (72.7%)
**AD‐specific risks**					
[^11^C]PiB SUVR^*^, ^‡^	1.1 (1.1, 1.2)	1.1 (1.0, 1.1)	1.1 (1.0, 1.1)	1.2 (1.1, 1.6)	1.1 (1.1, 1.2)
[^11^C]PiB positive^†^, ^‡^	29 (16.2%)	0 (0%)	5 (9.6%)	17 (36.2%)	7 (18.4%)
*APOE* ε4^†^	63 (31.3%)	19 (43.2%)	16 (28.6%)	16 (28.1%)	12 (27.3%)
**Composite UWMC (unitless)**					
Frontal lobe^*^	0.14 (0.09, 0.26)	0.10 (0.06, 0.14)^¶^	0.26 (0.15, 0.41)^¶^	0.09 (0.05, 0.13)^¶^	0.22 (0.14, 0.32)^¶^
Parietal lobe^*^	0.06 (0.01, 0.14)	0.02 (0.01, 0.06)^¶^	0.10 (0.02, 0.19)^¶^	0.04 (0.01, 0.06)^¶^	0.14 (0.04, 0.23)^¶^
Temporal lobe^*^	0.18 (0.10, 0.26)	0.16 (0.07, 0.21)^¶^	0.26 (0.20, 0.31)^¶^	0.11 (0.07, 0.18)^¶^	0.21 (0.11, 0.30)^¶^
Occipital lobe^*^	0.06 (0.01, 0.21)	0.03 (0.005, 0.12)^¶^	0.06 (0.02, 0.21)^¶^	0.06 (0.03, 0.22)	0.16 (0.04, 0.34)
**Composite WMH volume (unitless)** ^§^					
Frontal lobe^*^	55.3 (35.4, 111.1)	46.9 (30.8, 59.1)^¶^	103.2 (59.9, 236.9)^¶^	37.0 (19.1, 47.6)^¶^	99.9 (59.5, 150.9)^¶^
Parietal lobe^*^	7.9 (1.2, 30.5)	3.3 (0.69, 8.9)^¶^	21.4 (2.6, 39.2)^¶^	3.0 (0.24, 11.6)^¶^	30.5 (7.4, 50.8)^¶^
Temporal lobe^*^	29.5 (15.9, 48.9)	24.0 (11.7, 36.2)^¶^	51.7 (30.6, 74.1)^¶^	14.0 (8.6, 27.2)^¶^	37.3 (23.4, 51.4)^¶^
Occipital lobe^*^	2.8 (0.22, 13.6)	0.81 (0.06, 4.3)^¶^	3.6 (0.24, 11.8)^¶^	3.4 (0.16, 13.6)^¶^	10.5 (1.4, 25.0)^¶^

Abbreviations: AD, Alzheimer's disease; *APOE*, apolipoprotein E; CoBRA, Connectomics of Brain Aging; MCI, mild cognitive impairment; MoCA, Montreal Cognitive Assessment; PiB, Pittsburgh compound‐B; SUVR, standardized uptake value ratio; UWMC, unhealthy white matter connectivity; WMH, white matter hyperintensities.

* Median (25th percentile, 75th percentile).

^†^ *N* (%).

^‡^ *n* = 179.

^§^ Each lobar outcome (mm^3^) is normalized to intracranial volume (mm^3^), yielding unitless results.

^¶^ *P* < 0.05 for high UWMC vs. low UWMC comparison within a racialized group.

low global UWMC < median; high global UWMC ≥ median.

Participants with high global UWMC exhibited lower MoCA scores than those with low global UWMC (BA: median = 23.5 vs. 26.0, *P* = 0.002; nHW: median = 26.0 vs. 27.0, *P* = 0.02; Table 1). High global UWMC was also associated with more UWMC in the frontal (BA: median = 0.26 vs. 0.10, *P* < 0.0001; nHW: median = 0.22 vs. 0.09, *P* < 0.0001), parietal (BA: median = 0.10 vs. 0.02, *P* = 0.0002; nHW: median = 0.14 vs. 0.04, *P* < 0.0001), and temporal (BA: median = 0.06 vs. 0.03, *P* < 0.0001; nHW: median = 0.21 vs. 0.11, *P* = 0.0003) lobes as well as greater WMH volume in the frontal (BA: median = 103.2 vs. 46.9, *P* < 0.0001; nHW: median = 99.9 vs. 37.0, *P* < 0.0001), parietal (BA: median = 21.4 vs. 3.3, *P* = 0.0002; nHW: median = 30.5 vs. 3.0, *P* < 0.0001), temporal (BA: median = 51.7 vs. 24.0, *P* < 0.0001; nHW: median = 37.3 vs. 14.0, *P* < 0.0001), and occipital (BA: median = 3.6 vs. 0.81, *P* = 0.046; nHW: median = 10.5 vs. 3.4, *P* = 0.03) lobes in both racialized groups.

In BA, but not nHW, participants, a greater proportion of individuals with high global UWMC exhibited MCI than those with low global UWMC (41.1% vs. 9.1%, *P* = 0.0005, Table 1). High global UWMC was also associated with higher UWMC in the occipital lobe in BA participants (median = 0.06 vs. 0.03, *P* = 0.04). Conversely, among only nHW participants, those with high global UWMC were more likely to be female (77.3% vs. 50.9%, *P* = 0.006), had fewer years of education (median = 13.5 years vs. 14.0 years; *P* = 0.03), and exercised fewer hours per week (median = 9.9 vs. 13.0, *P* = 0.01) relative to those with low global UWMC.

Compared to those racialized as nHW, a higher proportion of BA participants were classified as having high versus low global UWMC (BA: 56.0%; nHW: 43.5%; Table 1), though this was not statistically significant (*P* = 0.08; Table S3 in supporting information). However, continuous global UWMC was greater in BA, versus nHW, participants (median = 0.21 vs. 0.17; *P* = 0.03; Table S3). BA participants also scored lower on MoCA than those who were nHW in both high (BA: median = 23.5; nHW: median = 26.0) and low (BA: median = 26.0; nHW: median = 27.0) global UWMC groups. MoCA scores among BA participants were lower than those in nHW participants overall (median = 24 vs. 27; *P* < 0.0001; Table S3). Compared to nHW participants, those racialized as BA exhibited lower global [^11^C]PiB SUVR (*P* < 0.0001) and no differences in *APOE* ε4 frequency (*P* = 0.26). Other between‐racialized group differences in participant characteristics are shown in Table S3.

### 3.1 Unhealthy white matter connectivity

#### 3.1.1 Aβ‐affected regions

In the data‐reduction step, 18 out of the 860 UWMC variables in Aβ‐affected regions were selected as being important predictors of MoCA score (Table 2). All but three of these UWMC variables had at least one end in a region within the frontal lobe.

**TABLE 2:** Associations of UWMC with MoCA score in Aβ‐affected regions.

Connecting regions					
Region A	Region B	Full sample β (*P* value)	Black American β (*P* value)	Non‐Hispanic White β (*P* value)	Interaction *P* value
**Frontal**	**Frontal**				
L caudal anterior cingulate cortex	L caudal middle frontal gyrus	−1.08 (0.006)^*^	−1.39 (0.01)^*^, ^†^	−0.92 (0.14)^†^	0.29
L superior frontal gyrus	R rostral middle frontal gyrus	−1.19 (0.0004)^*^	−1.20 (0.03)^*^	−1.25 (0.009)^*^	–
L rostral middle frontal gyrus	R caudal anterior cingulate cortex	−1.09 (0.002)^*^	−0.96 (0.06)^†^	−1.31 (0.01)^*^, ^†^	0.75
L lateral orbitofrontal gyrus	L rostral anterior cingulate cortex	−1.08 (0.0009)^*^	−1.36 (0.004)^*^, ^†^	−1.04 (0.04)^†^	0.80
L pars orbitalis	R caudal anterior cingulate cortex	−1.01 (0.008)^*^	−0.89 (0.09)^†^	−1.37 (0.03)^*^, ^†^	0.74
**Frontal**	**Parietal**				
L frontal pole	R supramarginal gyrus	−2.44 (0.02)^*^	−2.55 (0.01)^*^	NA^‡^	–
R superior frontal gyrus	R inferior parietal gyrus	−1.52 (< 0.0001)^*^	−1.69 (0.0002)^*^	−1.41 (0.005)^*^	–
L caudal middle frontal gyrus	R precuneus	−1.36 (0.0003)^*^	−0.73 (0.16)^†^	−2.42 (< 0.0001)^*^, ^†^	0.12
R pars orbitalis	R precuneus	−1.34 (0.0007)^*^	−1.04 (0.06)^†^	−1.97 (0.002)^*^, ^†^	0.25
**Frontal**	**Striatum**				
L rostral middle frontal gyrus	R anterior ventral striatum	−1.24 (0.005)^*^	−1.01 (0.11)^†^	−1.58 (0.02)^*^, ^†^	0.44
L pars triangularis	R anterior ventral striatum	−0.55 (0.32)	0.53 (0.57)	−1.33 (0.07)	–
**Frontal**	**Temporal**				
L rostral anterior cingulate gyrus	R banks of superior temporal sulcus	−2.03 (0.02)^*^	−1.09 (0.35)	−2.60 (0.05)	–
R superior frontal gyrus	R superior temporal gyrus	−1.55 (< 0.0001)^*^	−1.84 (< 0.0001)^*^	−1.47 (0.0004)^*^	–
R caudal middle frontal gyrus	R banks of superior temporal sulcus	−1.32 (0.006)^*^	−1.15 (0.09)^†^	−1.73 (0.02)^*^, ^†^	0.81
R caudal middle frontal gyrus	R superior temporal gyrus	−1.24 (0.001)^*^	−1.24 (0.03)^†^	−1.61 (0.004)^*^, ^†^	0.78
**Parietal**	**Insula**				
R isthmus cingulate cortex	L insula	−0.88 (0.02)^*^	−0.68 (0.18)	−1.23 (0.04)	–
**Parietal**	**Striatum**				
L isthmus cingulate cortex	R anterior ventral striatum	−3.03 (< 0.0001)^*^	−2.01 (0.08)^†^	−3.57 (< 0.0001)^*^, ^†^	0.18
**Temporal**	**Insula**				
R banks of superior temporal sulcus	R insula	−1.24 (0.0005)^*^	−1.04 (0.04)^†^	−1.81 (0.0007)^*^, ^†^	0.55

Abbreviations: Aβ, beta‐amyloid; AD, Alzheimer's disease; *APOE*, apolipoprotein E; FDR, false discovery rate; MoCA, Montreal Cognitive Assessment; UWMC, unhealthy white matter connectivity.

* FDR‐corrected *P* value < 0.05, uncorrected *P* values shown.

^†^ Relationship significant in one racialized group, but not the other, so an UWMC‐by‐racialized group interaction term was tested in the full sample.

^‡^ All participants had UWMC = 0.

Models adjusted for age, sex, racialized group (full sample only), education, physical activity, diabetes, hypertension, high cholesterol, and *APOE* ε4.

In individual robust regression models, we found that UWMC was significantly associated with lower MoCA score in 17 of the 18 pairs of connecting regions (FDR‐corrected *P* < 0.05; Table 2), 14 of which included at least one end in the frontal lobe. Of the significant relationships, UWMC between the left isthmus cingulate cortex and right anterior ventral striatum, left frontal pole and right supramarginal gyrus, and left rostral anterior cingulate gyrus and right banks of the superior temporal sulcus were most strongly associated with MoCA score, where a 1% higher UWMC was respectively associated with a 0.030‐, 0.024‐, and 0.020‐point lower MoCA score. In other pairwise connections that were significantly related to MoCA score, 1% greater UWMC was related to MoCA score ranging from 0.009 to 0.016 points lower.

When stratified by racialized group, UWMC was inversely associated with MoCA score for both BA and nHW participants in 3 out of the 18 pairs of connecting regions (FDR‐corrected *P* < 0.05; Table 2): the left superior frontal gyrus and right rostral middle frontal gyrus, the right superior frontal gyrus and right inferior parietal gyrus, and the right superior frontal gyrus and right superior temporal gyrus. Of these, UWMC between the right superior frontal and right superior temporal gyri was most strongly related to MoCA score in both racialized groups; 1% higher UWMC between these regions was associated with a MoCA score that was 0.018 points lower among BA participants and 0.015 points lower in nHW participants. Further, 1% greater UWMC between the two other regions was related to a MoCA score that ranged from 0.012 to 0.017 points lower for both groups.

In BA, but not nHW, participants, higher UWMC was related to lower MoCA score between 3 of the 18 pairwise connections (FDR‐corrected *P* < 0.05; Table 2). In this group, 1% greater UWMC between the left caudal anterior cingulate cortex and left caudal middle frontal gyrus and between the left lateral orbitofrontal gyrus and left rostral anterior cingulate cortex were both associated with a 0.014‐point lower MoCA score.

Conversely, in nHW, but not BA, participants, higher UWMC was associated with lower MoCA scores in 8 out of the 18 pairwise connections (FDR‐corrected *P* < 0.05; Table 2). Among nHW participants, UWMC between the left isthmus cingulate cortex and right anterior ventral striatum was most strongly associated with MoCA score, with 1% higher UWMC between these two regions related to a 0.036‐point lower MoCA score. UWMC between the left rostral anterior cingulate gyrus and right banks of the superior temporal sulcus and between the left caudal middle frontal gyrus and right precuneus were also strongly related to MoCA score; 1% greater UWMC on these connections was related to a MoCA score that was 0.026 and 0.024 points lower, respectively. Of the remaining pairwise connections that were significantly related to MoCA score in nHW participants, higher UWMC by 1% was related to MoCA scores that ranged from 0.009 to 0.0197 points lower.

In the full sample, racialization did not modify the relationship between any UWMC and MoCA (*P* > 0.05 for all interaction terms; Table 2).

#### 3.1.2 Tau‐affected regions

In the data‐reduction step, 8 of the 65 UWMC variables in tau‐affected regions were selected as being most associated with MoCA score (Table 3).

**TABLE 3:** Associations of UWMC with MoCA score in tau‐affected regions.

Connecting regions					
Region A	Region B	Full sample β (*P* value)	Black American β (*P* value)	Non‐Hispanic White β (*P* value)	Interaction *P* value
L inferior temporal gyrus	R parahippocampal gyrus	−2.26 (0.0008)^*^	−2.40 (0.001)^*^	−1.98 (0.01)^*^	–
L fusiform	R entorhinal cortex	−1.89 (0.0003)^*^	−1.85 (0.007)^*^	−2.22 (0.01)^*^	–
L amygdala	L fusiform gyrus	−0.97 (0.008)^*^	−0.98 (0.06)^†^	−1.43 (0.01)^*^, ^†^	0.94
L amygdala	L inferior temporal gyrus	−0.95 (0.006)^*^	−0.76 (0.12)^†^	−1.39 (0.01)^*^, ^†^	0.38
L inferior temporal gyrus	L parahippocampal gyrus	−0.66 (0.06)	−0.30 (0.54)	−0.91 (0.08)	–
L fusiform	L middle temporal gyrus	−0.64 (0.03)^*^	−0.14 (0.73)^†^	−1.37 (0.003)^*^, ^†^	0.15
L fusiform	L parahippocampal gyrus	−0.46 (0.13)	0.39 (0.39)^†^	−1.14 (0.009)^*^, ^†^	0.06
L amygdala	R fusiform gyrus	0.23 (0.09)	0.33 (0.06)	0.14 (0.52)	–

Abbreviations: *APOE*, apolipoprotein E; FDR, false discovery rate; MoCA, Montreal Cognitive Assessment; UWMC, unhealthy white matter connectivity.

* FDR‐corrected *P* value < 0.05, uncorrected *P* values shown.

^†^ Relationship significant in one racialized group, but not the other, so an UWMC‐by‐racialized group interaction term was tested in the full sample.

Models adjusted for age, sex, racialized group (full sample only), education, physical activity, diabetes, hypertension, high cholesterol, and *APOE* ε4.

In individual robust regression models, higher UWMC was significantly associated with lower MoCA score in five of the eight selected connections (FDR‐corrected *P* < 0.05; Table 3). The strongest of these relationships was UWMC between the left inferior temporal gyrus and right parahippocampal gyrus, wherein 1% higher UWMC was associated with a MoCA score that was 0.023 points lower. Among the other connections that were significantly related to MoCA score, 1% greater UWMC was related to a MoCA score that ranged from 0.006 to 0.019 points lower.

In racialized group‐stratified analyses, higher UWMC was associated with lower MoCA scores for both BA and nHW participants in two of the eight pairwise connections (FDR‐corrected *P* < 0.05; Table 3). Between the left inferior temporal and right parahippocampal gyri, 1% greater UWMC was associated with a 0.024‐ and 0.020‐point lower MoCA score among BA and nHW participants, respectively. A 1% higher UWMC between the left fusiform and right entorhinal cortex was associated with MoCA score that was 0.019 points lower in BA participants and 0.022 points lower in nHW participants.

Among nHW, but not BA, participants, UWMC was inversely associated with MoCA score in four of the eight pairs of connecting regions (FDR‐corrected *P* < 0.05; Table 3): the left amygdala and left fusiform gyrus, the left amygdala and left inferior temporal gyrus, the left fusiform and left middle temporal gyrus, and the left fusiform and left parahippocampal gyrus. These relationships were fairly similar in strength, wherein a 1% greater UWMC was associated with MoCA score that ranged from 0.012 to 0.014 points lower.

Interactions between racialization and UWMC on MoCA score were not significant in the full sample (*P* > 0.05 for all interaction terms; Table 3).

#### 3.1.3 Lobar unhealthy white matter connectivity and white matter hyperintensity volume

Lobar UWMC and WMH volume were highly correlated in the full sample for the frontal (*rho* = 0.80, *P* < 0.0001), parietal (*rho* = 0.88, *P* < 0.0001), and occipital lobes (*rho* = 0.87, *P* < 0.0001). While statistically significant, the correlation between temporal lobe UWMC and WMH volume were comparatively not as well correlated (*rho* = 0.43, *P* < 0.0001).

In the full sample, greater UWMC was associated with lower MoCA score in the frontal and parietal lobes (*P* ≤ 0.047; Table 4). When stratified by racialized group, UWMC in the frontal lobe was inversely associated with MoCA score in both BA and nHW (*P* ≤ 0.049) participants. Greater UWMC in the parietal lobe was associated with lower MoCA score in nHW (*P* = 0.03), but not BA (*P* = 0.44), participants. The interaction between racialization and parietal UWMC on MoCA score was not significant.

**TABLE 4:** Associations of lobar UWMC and lobar WMH volume with MoCA score.

	Full sample	Black American	Non‐Hispanic White	Interaction
	β (*P* value)	β (*P* value)	β (*P* value)	*P* value
**Lobar UWMC**				
Frontal	−1.55 (0.006)^*^	−1.73 (0.03)^*^	−1.75 (0.049)^*^	–
Parietal	−0.48 (0.047)^*^	−0.26 (0.44)^†^	−0.84 (0.03)^*^, ^†^	0.506
Temporal	−0.75 (0.22)	−0.87 (0.30)	−0.77 (0.44)	–
Occipital	−0.16 (0.51)	0.03 (0.94)	‐0.35 (0.32)	–
**Lobar WMH volume**				
Frontal	−1.98 (0.0003)^*^	−1.66 (0.04)^*^	−2.55 (0.001)^*^	–
Parietal	−0.64 (0.002)^*^	−0.64 (0.047)^*^	−0.73 (0.01)^*^	–
Temporal	−1.27 (0.004)^*^	−1.01 (0.10)^†^	−2.00 (0.008)^*^, ^†^	0.54
Occipital	−0.19 (0.36)	−0.12 (0.70)	−0.35 (0.25)	–

Abbreviations: *APOE*, apolipoprotein E; MoCA Montreal Cognitive Assessment; UWMC, unhealthy white matter connectivity; WMH, white matter hyperintensities.

* *P* value < 0.05.

^†^ Relationship significant in one racialized group, but not the other, so an UWMC‐by‐racialized group interaction term was tested in the full sample.

Models adjusted for age, sex, racialized group (full sample only), education, physical activity, diabetes, hypertension, high cholesterol, and *APOE* ε4.

Greater WMH volume was associated with lower MoCA score in the full sample in the frontal, parietal, and temporal (*P* ≤ 0.004) lobes (Table 4). In racialized group‐stratified analyses, WMH volume in the frontal (BA: *P* = 0.04; nHW: *P* = 0.001) and parietal (BA: *P* = 0.047; nHW: *P* = 0.01) lobes was inversely associated with MoCA score in both racialized groups. In nHW, but not BA, participants, greater WMH volume was related to lower MoCA score in the temporal (BA: *P* = 0.10; nHW: *P* = 0.0008) lobe. In the full sample, racialization did not modify the relationship between any lobar WMH volume and MoCA score.

## 4 DISCUSSION

We found (1) greater UWMC burden in BA versus nHW participants, (2) inverse relationships between UWMC in AD pathology‐affected regions and MoCA score, and (3) no racialized group differences in associations of UWMC with MoCA score. To our knowledge, this is the first study to measure UWMC and its relationship to cognition. Others found that WMH on association and projection fibers influences domain‐specific cognition in older adults.^45^ Our study builds upon these findings by localizing lesions on connectome‐derived fibers.

### 4.1 Unhealthy white matter connectivity in Aβ‐affected regions and cognition

We identified relationships between UWMC in Aβ pathology–affected regions and cognition. The strongest of these were on connections between the left isthmus cingulate cortex and right anterior ventral striatum, the left frontal pole and right supramarginal gyrus, and the left rostral anterior cingulate gyrus and right banks of the superior temporal sulcus. While effect sizes were small, we examined differences in MoCA score associated with 1% greater UWMC on one pairwise connection. If greater UWMC was present across numerous connections (as is likely, given the diffusivity of WMH), the impact on cognition may have been stronger. The small magnitude of the relationships may also be due to most participants being cognitively unimpaired. Studies with more clinically diverse participants should investigate the role of UWMC on these and surrounding pairwise connections in cognition.

Previous work found that posterior WMH is associated with global^46^ and domain‐specific^47^ cognitive test performance, suggesting that WMH and Aβ may additively or interactively influence cognition. We found most Aβ‐affected connections associated with MoCA score included at least one region in the frontal, rather than parietal or occipital, lobe. We may not have detected relationships in more posterior regions given the low number of participants with MCI or AD, in whom posterior WMH are more severe^48^ and more strongly associated with cognition, compared to those in earlier stages of cognitive impairment.^46^ The predominance of associations between frontal UWMC and MoCA score also suggests that etiology of spatially distinct WMH may differ. Parietal and occipital WMH are thought to be associated with Wallerian degeneration due to Aβ burden, whereas frontal WMH are related to hypoperfusion caused by vascular risks.^49^, ^50^ The paucity of significant associations between posterior UWMC and MoCA score, partnered with the low Aβ burden in our sample, is in line with this. As such, our findings likely reflect independent vascular effects of UWMC on cognition. Whether frontal UWMC also influences co‐localized Aβ in later disease stages, as has been hypothesized,^51^, ^52^, ^53^, ^54^ and cognitive impairment, should be studied in future work.

### 4.2 Unhealthy white matter connectivity in tau‐affected regions and cognition

We also detected associations between UWMC in tau‐affected regions and cognition, the strongest of which were on connections between the left inferior temporal and right parahippocampal gyrus and the left fusiform and right entorhinal cortex. As with findings in Aβ‐affected regions, effect sizes were small, likely due to the same reasons listed above: that we examined differences in MoCA score per 1% higher UWMC on single pairwise connections and most participants were cognitively unimpaired.

We are aware of one other study that found that lobar temporal WMH was associated with cognition in AD participants.^46^ Our work builds upon this by identifying this relationship in regions that are specifically affected by tau pathology.

Despite not collecting tau data, we can infer minimal pathology as participants exhibited low [^11^C]PiB SUVRs and most were cognitively unimpaired.^55^ Thus, observed relationships between medial temporal UWMC and cognition are likely due to independent effects of WMH, rather than interactions with tau. Notably, most connections included regions in later, rather than earlier, Braak stages.^56^ Others found associations of parietal WMH with tau accumulation and incident clinical AD,^57^ perhaps through white matter injury and resultant hyperphosphorylation.^13^ Our findings suggest that the role of medial temporal WMH on tau accumulation and cognition should be further investigated.

### 4.3 No racialized group differences in associations between unhealthy white matter connectivity and cognition

Relative to nHW participants, BA individuals exhibited more UWMC and lower MoCA scores. These findings are consistent with reports of racialized group differences in global and regional WMH burden^4^, ^5^, ^6^ and global neuropsychological test performance.^58^, ^59^ UWMC‐and‐MoCA score associations were more often significant in nHW, versus BA, participants for both Aβ‐ and tau‐affected regions. However, [^11^C]PiB SUVRs were higher in nHW participants relative to those who were BA, indicating more advanced pathological disease. Thus, differential AD pathology, which can affect both WMH and cognition, may drive this difference. BA racialization did not modify any UWMC‐and‐MoCA score associations. This is in contrast to at least two other studies that reported a stronger relationship between WMH volume and cognitive impairment in BA, versus nHW, participants.^7^, ^8^ However, these studies may not be comparable to ours; both quantified WMH as a global measure and one reported similar WMH burden between BA and nHW participants.^7^

Using our UWMC method, our results suggest that WMH in AD pathology‐affected regions may partially drive clinical AD disparities between BA and nHW populations through differences in prevalence of WMH in these regions, rather than through differences in UWMC/WMH–cognition relationships.^60^ As such, intervening on upstream risk factors for UWMC/WMH may reduce racialized group disparities in clinical AD.^60^ Individual‐level interventions that target vascular risk factors, including intensive blood pressure reduction, statin usage, and lifestyle modifications (e.g., exercise), have been shown to slow progression of WMH,^3^ but may not be feasible for those who lack access to necessary resources. Other interventions, particularly at institutional and structural levels, should be investigated. BA individuals are more likely than those who are nHW to experience systemic and interpersonal racism in the forms of social, environmental, and economic adversity.^61^ Cumulative exposure to these stressors leads to vascular risk factors^62^, ^63^, ^64^ associated with WMH. Despite this, studies examining contextual exposures in relation to WMH and clinical/pathological AD are sparse. Further interrogation of relationships and mechanisms between lived experiences and WMH/UWMC is necessary to develop appropriate interventions.

### 4.4 Unhealthy white matter connectivity as a measure of white matter hyperintensities

Lobar UWMC was highly correlated with WMH volume in the frontal, parietal, and occipital lobes. Both composite UWMC and WMH volume in these lobes were inversely associated with MoCA score among the full sample and racialized group‐stratified analyses. Together, these results suggest that UWMC is a valid measure of white matter pathology, which is a key strength of this work. Relative to composite WMH outcomes, the increased spatial precision of UWMC affords the opportunity to more closely investigate WMH, specifically those co‐localized with AD pathology, throughout the natural history of clinical AD. For example, using our method, investigators will be positioned to examine (1) relationships of UWMC in AD pathology‐affected regions with Aβ and tau deposition and accumulation and (2) interactions between AD pathology and co‐localized UWMC on cross‐sectional and longitudinal cognition. This should contribute to understanding the role of WMH in pathological and clinical AD.

Temporal lobe UWMC and WMH volume were not as well correlated. We also detected an inverse relationship between temporal lobe WMH volume and MoCA score, but not temporal lobe UWMC and MoCA score. These discrepant findings may be due to FreeSurfer parcellation quality in the temporal regions, which are not always perfectly parcellated as they are small and subject to neurodegeneration in older adults. While we visually inspected FreeSurfer atlases prior to analysis, it is possible that minute inaccuracies may drive between‐method differences. Still, because our UWMC results were consistent with WMH volume in other lobes and current scientific literature, we believe that temporal lobe UWMC is a valid, albeit less sensitive, measure of white matter pathology than in other lobes. Ongoing efforts to optimize parcellation methods should improve its utility in future work.

### 4.5 Limitations

This study has limitations. First, the cross‐sectional design limits our ability to infer temporality and causality. Second, while we attempted to prevent this in our analysis pipeline, tractography can produce anatomically incorrect connections.^65^ Additionally, other vascular risk factors that influence both WMH and cognition, like body mass index and smoking history, were not included in analyses. Finally, we excluded individuals with contraindication for MRI and history of stroke, the latter of which likely limited the range of UWMC severity in this dataset. Selection bias conferred by these criteria may partially drive the counterintuitive distributions by global UWMC; younger age and lower prevalence of cardiovascular risk factors were non‐significantly associated with high global UWMC in nHW and BA participants, respectively.

## 5 CONCLUSIONS

Our results contribute to the growing body of literature implicating WMH in clinical AD. While BA participants exhibited lower MoCA scores and more severe UWMC, racialization did not modify relationships between UWMC and cognition. Thus, comparatively greater WMH/UWMC burden in AD pathology–affected regions, but not stronger WMH/UWMC‐and‐cognition associations, may partially explain greater clinical AD burden in BA versus nHW populations. Interventions that reduce WMH may minimize clinical AD disparities, but more work is necessary to characterize drivers of WMH/UWMC burden, particularly at structural and institutional levels.

## CONFLICT OF INTEREST STATEMENT

The authors have nothing to report. Author disclosures are available in the supporting information.

## CONSENT STATEMENT

All human participants provided informed consent.

## Supporting information

Supporting information

Supporting information

## References

[1] Rajan KB , Weuve J , Barnes LL , Wilson RS , Evans DA . Prevalence and incidence of clinically diagnosed Alzheimer's disease dementia from 1994 to 2012 in a population study. Alzheimers Dement. 2019;15:1‐7. doi:10.1016/j.jalz.2018.07.216 30195482 PMC6531287

[2] Mayeda ER , Glymour MM , Quesenberry CP , Whitmer RA . Inequalities in dementia incidence between six racial and ethnic groups over 14 years. Alzheimers Dement. 2016;12:216‐224. doi:10.1016/j.jalz.2015.12.007 26874595 PMC4969071

[3] Wardlaw JM , Smith C , Dichgans M . Small vessel disease: mechanisms and clinical implications. Lancet Neurol. 2019;18:684‐696. doi:10.1016/S1474-4422(19)30079-1 31097385

[4] Royse SK , Cohen AD , Snitz BE , Rosano C . Differences in alzheimer's disease and related dementias pathology among african american and hispanic women: a qualitative literature review of biomarker studies. Front Syst Neurosci. 2021;15:685957. doi:10.3389/fnsys.2021.685957 34366799 PMC8334184

[5] Turney IC , Lao PJ , Rentería MA , et al. Brain aging among racially and ethnically diverse middle‐aged and older adults. JAMA Neurol. 2023;80:73‐81. doi:10.1001/jamaneurol.2022.3919 36374494 PMC9664371

[6] Morrison C , Dadar M , Manera AL , Collins DL . Racial differences in white matter hyperintensity burden in older adults. Neurobiol Aging. 2023;122:112‐119. doi:10.1016/j.neurobiolaging.2022.11.012 36543016

[7] Howell JC , Watts KD , Parker MW , et al. Race modifies the relationship between cognition and Alzheimer's disease cerebrospinal fluid biomarkers. Alzheimers Res Ther. 2017;9:88. doi:10.1186/s13195-017-0315-1 29096697 PMC5668981

[8] Zahodne LB , Manly JJ , Narkhede A , et al. Structural MRI predictors of late‐life cognition differ across African Americans, Hispanics, and Whites. Curr Alzheimer Res. 2015;12:632‐639. doi:10.2174/1567205012666150530203214 26027808 PMC4872300

[9] Attems J , Jellinger KA . The overlap between vascular disease and Alzheimer's disease–lessons from pathology. BMC Med. 2014;12:206. doi:10.1186/s12916-014-0206-2 25385447 PMC4226890

[10] Kapasi A , DeCarli C , Schneider JA . Impact of multiple pathologies on the threshold for clinically overt dementia. Acta Neuropathol. 2017;134:171‐186. doi:10.1007/s00401-017-1717-7 28488154 PMC5663642

[11] Friston KJ . The disconnection hypothesis. Schizophr Res. 1998;30:115‐125. doi:10.1016/s0920-9964(97)00140-0 9549774

[12] Weller RO , Subash M , Preston SD , Mazanti I , Carare RO . Perivascular drainage of amyloid‐beta peptides from the brain and its failure in cerebral amyloid angiopathy and Alzheimer's disease. Brain Pathol. 2008;18:253‐266. doi:10.1111/j.1750-3639.2008.00133.x 18363936 PMC8095597

[13] Laing KK , Simoes S , Baena‐Caldas GP , et al. Cerebrovascular disease promotes tau pathology in Alzheimer's disease. Brain Commun. 2020;2:fcaa132. doi:10.1093/braincomms/fcaa132 33215083 PMC7660042

[14] Hawkes CA , Jayakody N , Johnston DA , Bechmann I , Carare RO . Failure of perivascular drainage of β‐amyloid in cerebral amyloid angiopathy. Brain Pathol. 2014;24:396‐403. doi:10.1111/bpa.12159 24946077 PMC8029317

[15] Langen CD , Cremers LGM , de Groot M , et al. Disconnection due to white matter hyperintensities is associated with lower cognitive scores. Neuroimage. 2018;183:745‐756. doi:10.1016/j.neuroimage.2018.08.037 30144572

[16] Cohen AD , Bruña R , Chang Y‐F , et al. Connectomics in brain aging and dementia—the background and design of a study of a connectome related to human disease. Front Aging Neurosci. 2021;13:669490. doi:10.3389/fnagi.2021.669490 34690734 PMC8530182

[17] Newman AB , Glynn NW , Taylor CA , et al. Health and function of participants in the Long Life Family Study: a comparison with other cohorts. Aging (Albany, NY). 2011;3:63‐76. doi:10.18632/aging.100242 21258136 PMC3047140

[18] Bambs C , Kip KE , Dinga A , Mulukutla SR , Aiyer AN , Reis SE . Low prevalence of “ideal cardiovascular health” in a community‐based population: the heart strategies concentrating on risk evaluation (Heart SCORE) study. Circulation. 2011;123:850‐857. doi:10.1161/CIRCULATIONAHA.110.980151 21321154 PMC3061396

[19] Klunk WE , Engler H , Nordberg A , et al. Imaging brain amyloid in Alzheimer's disease with Pittsburgh Compound‐B. Ann Neurol. 2004;55:306‐319. doi:10.1002/ana.20009 14991808

[20] Snitz BE , Tudorascu DL , Yu Z , et al. Associations between NIH Toolbox Cognition Battery and in vivo brain amyloid and tau pathology in non‐demented older adults. Alzheimers Dement (Amst). 2020;12:e12018. doi:10.1002/dad2.12018 32426450 PMC7228102

[21] Jenkinson M , Smith S . A global optimisation method for robust affine registration of brain images. Med Image Anal. 2001;5:143‐156. doi:10.1016/s1361-8415(01)00036-6 11516708

[22] FreeSurfe Fischl . FreeSurfer. Neuroimage. 2012;62:774‐781. doi:10.1016/j.neuroimage.2012.01.021 22248573 PMC3685476

[23] Tziortzi AC , Searle GE , Tzimopoulou S , et al. Imaging dopamine receptors in humans with [11C]‐(+)‐PHNO: dissection of D3 signal and anatomy. Neuroimage. 2011;54:264‐277. doi:10.1016/j.neuroimage.2010.06.044 20600980

[24] Wu M , Fatukasi O , Yang S , et al. HIV disease and diabetes interact to affect brain white matter hyperintensities and cognition. AIDS. 2018;32:1803‐1810. doi:10.1097/QAD.0000000000001891 29794829 PMC6082131

[25] Glasser MF , Sotiropoulos SN , Wilson JA , et al. The minimal preprocessing pipelines for the Human Connectome Project. Neuroimage. 2013;80:105‐124. doi:10.1016/j.neuroimage.2013.04.127 23668970 PMC3720813

[26] Yeh F‐C , Verstynen TD , Wang Y , Fernández‐Miranda JC , Tseng W‐YI . Deterministic diffusion fiber tracking improved by quantitative anisotropy. PLoS One. 2013;8:e80713. doi:10.1371/journal.pone.0080713 24348913 PMC3858183

[27] Yeh F‐C , Tseng W‐YI . NTU‐90: a high angular resolution brain atlas constructed by q‐space diffeomorphic reconstruction. Neuroimage. 2011;58:91‐99. doi:10.1016/j.neuroimage.2011.06.021 21704171

[28] Yeh F‐C , Panesar S , Barrios J , et al. Automatic removal of false connections in diffusion mri tractography using topology‐informed pruning (TIP). Neurotherapeutics. 2019;16:52‐58. doi:10.1007/s13311-018-0663-y 30218214 PMC6361061

[29] Jack CR , Wiste HJ , Weigand SD , et al. Defining imaging biomarker cut points for brain aging and Alzheimer's disease. Alzheimers Dement. 2017;13:205‐216. doi:10.1016/j.jalz.2016.08.005 27697430 PMC5344738

[30] Fonov VS , Evans AC , Botteron K , et al. Unbiased average age‐appropriate atlases for pediatric studies. Neuroimage. 2011;54:313‐327. doi:10.1016/j.neuroimage.2010.07.033 20656036 PMC2962759

[31] Nasreddine ZS , Phillips NA , Bédirian V , et al. The Montreal Cognitive Assessment, MoCA: a brief screening tool for mild cognitive impairment. J Am Geriatr Soc. 2005;53:695‐699. doi:10.1111/j.1532-5415.2005.53221.x 15817019

[32] Kalewold KH . Race and medicine in light of the new mechanistic philosophy of science. Biol Philos. 2020;35:41. doi:10.1007/s10539-020-09759-x

[33] Lopez OL , Becker JT , Klunk W , et al. Research evaluation and diagnosis of possible Alzheimer's disease over the last two decades: iI. Neurology. 2000;55:1863‐1869. doi:10.1212/wnl.55.12.1863 11134386

[34] Manly JJ , Jacobs DM , Touradji P , Small SA , Stern Y . Reading level attenuates differences in neuropsychological test performance between African American and White elders. J Int Neuropsychol Soc. 2002;8:341‐348. doi:10.1017/s1355617702813157 11939693

[35] Albert MS , DeKosky ST , Dickson D , et al. The diagnosis of mild cognitive impairment due to Alzheimer's disease: recommendations from the National Institute on Aging‐Alzheimer's Association workgroups on diagnostic guidelines for Alzheimer's disease. Alzheimers Dement. 2011;7:270‐279. doi:10.1016/j.jalz.2011.03.008 21514249 PMC3312027

[36] McKhann GM , Knopman DS , Chertkow H , et al. The diagnosis of dementia due to Alzheimer's disease: recommendations from the National Institute on Aging‐Alzheimer's Association workgroups on diagnostic guidelines for Alzheimer's disease. Alzheimers Dement. 2011;7:263‐269. doi:10.1016/j.jalz.2011.03.005 21514250 PMC3312024

[37] Cohen AD , Mowrey W , Weissfeld LA , et al. Classification of amyloid‐positivity in controls: comparison of visual read and quantitative approaches. Neuroimage. 2013;71:207‐215. doi:10.1016/j.neuroimage.2013.01.015 23353602 PMC3605888

[38] Greenland S , Pearl J , Robins JM . Causal diagrams for epidemiologic research. Epidemiology. 1999;10:37‐48. doi:10.1097/00001648-199901000-00008 9888278

[39] Stewart AL , Mills KM , King AC , Haskell WL , Gillis D , Ritter PL . CHAMPS physical activity questionnaire for older adults: outcomes for interventions. Med Sci Sports Exerc. 2001;33:1126‐1141. doi:10.1097/00005768-200107000-00010 11445760

[40] Kamboh MI , Aston CE , Hamman RF . The relationship of APOE polymorphism and cholesterol levels in normoglycemic and diabetic subjects in a biethnic population from the San Luis Valley, Colorado. Atherosclerosis. 1995;112:145‐159. doi:10.1016/0021-9150(94)05409-c 7772075

[41] Hayes‐Larson E , Kezios KL , Mooney SJ , Lovasi G . Guidelines for a useful Table 1. J Clin Epidemiol. 2019;114:125‐132. doi:10.1016/j.jclinepi.2019.06.011. Who is in this study, anyway?. 31229583 PMC6773463

[42] Mayeli A , Wilson JD , Donati FL , LaGoy AD , Ferrarelli F . Sleep spindle alterations relate to working memory deficits in individuals at clinical high‐risk for psychosis. Sleep. 2022;45. doi:10.1093/sleep/zsac193 PMC9644126 35981865

[43] Benjamini Y , Hochberg Y . Controlling the false discovery rate: a practical and powerful approach to multiple testing. J Royal Statist Soci: Series B (Methodolog). 1995;57:289‐300. doi:10.1111/j.2517-6161.1995.tb02031.x

[44] Vable AM , Diehl SF , Glymour MM . Code review as a simple trick to enhance reproducibility, accelerate learning, and improve the quality of your team's research. Am J Epidemiol. 2021;190:2172‐2177. doi:10.1093/aje/kwab092 33834188

[45] Rizvi B , Lao PJ , Colón J , et al. Tract‐defined regional white matter hyperintensities and memory. Neuroimage Clin. 2020;25:102143. doi:10.1016/j.nicl.2019.102143 31887716 PMC6939088

[46] Garnier‐Crussard A , Bougacha S , Wirth M , et al. White matter hyperintensity topography in Alzheimer's disease and links to cognition. Alzheimers Dement. 2022;18:422‐433. doi:10.1002/alz.12410 34322985 PMC9292254

[47] Huynh K , Piguet O , Kwok J , et al. Clinical and biological correlates of white matter hyperintensities in patients with behavioral‐variant frontotemporal dementia and Alzheimer disease. Neurology. 2021;96:e1743‐e1754. doi:10.1212/WNL.0000000000011638 33597290

[48] Brickman AM . Contemplating Alzheimer's disease and the contribution of white matter hyperintensities. Curr Neurol Neurosci Rep. 2013;13:415. doi:10.1007/s11910-013-0415-7 24190781 PMC3874404

[49] Pålhaugen L , Sudre CH , Tecelao S , et al. Brain amyloid and vascular risk are related to distinct white matter hyperintensity patterns. J Cereb Blood Flow Metab. 2021;41:1162‐1174. doi:10.1177/0271678X20957604 32955960 PMC8054718

[50] McAleese KE , Walker L , Graham S , et al. Parietal white matter lesions in Alzheimer's disease are associated with cortical neurodegenerative pathology, but not with small vessel disease. Acta Neuropathol. 2017;134:459‐473. doi:10.1007/s00401-017-1738-2 28638989 PMC5563333

[51] Honjo K , Black SE , Verhoeff NPLG . Alzheimer's Disease, Cerebrovascular Disease, and the β‐amyloid Cascade. Can J Neurol Sci. 2012;39:712‐728. doi:10.1017/S0317167100015547 23227576

[52] de la Torre JC . Critically attained threshold of cerebral hypoperfusion: the CATCH hypothesis of Alzheimer's pathogenesis. Neurobiol Aging. 2000;21:331‐342. doi:10.1016/s0197-4580(00)00111-1 10867218

[53] Iturria‐Medina Y , Sotero RC , Toussaint PJ , Mateos‐Pérez JM , Evans AC . Alzheimer's Disease Neuroimaging Initiative. Early role of vascular dysregulation on late‐onset Alzheimer's disease based on multifactorial data‐driven analysis. Nat Commun. 2016;7:11934. doi:10.1038/ncomms11934 27327500 PMC4919512

[54] Thal DR . The pre‐capillary segment of the blood‐brain barrier and its relation to perivascular drainage in Alzheimer's disease and small vessel disease. Sci World J. 2009;9:557‐563. doi:10.1100/tsw.2009.72 PMC5823174 19578713

[55] Jack CR , Holtzman DM . Biomarker modeling of Alzheimer's disease. Neuron. 2013;80:1347‐1358. doi:10.1016/j.neuron.2013.12.003 24360540 PMC3928967

[56] Braak H , Braak E . Neuropathological stageing of Alzheimer‐related changes. Acta Neuropathol. 1991;82:239‐259. doi:10.1007/BF00308809 1759558

[57] Tosto G , Zimmerman ME , Hamilton JL , Carmichael OT , Brickman AM . Alzheimer's Disease Neuroimaging Initiative. The effect of white matter hyperintensities on neurodegeneration in mild cognitive impairment. Alzheimers Dement. 2015;11:1510‐1519. doi:10.1016/j.jalz.2015.05.014 26079417 PMC4677059

[58] Gross AL , Mungas DM , Crane PK , et al. Effects of education and race on cognitive decline: an integrative study of generalizability versus study‐specific results. Psychol Aging. 2015;30:863‐880. doi:10.1037/pag0000032 26523693 PMC4679562

[59] Weuve J , Barnes LL , Mendes de Leon CF , et al. Cognitive aging in black and white americans: cognition, cognitive decline, and incidence of alzheimer disease dementia. Epidemiology. 2018;29:151‐159. doi:10.1097/EDE.0000000000000747 28863046 PMC5718953

[60] Ward JB , Gartner DR , Keyes KM , Fliss MD , McClure ES , Robinson WR . How do we assess a racial disparity in health? Distribution, interaction, and interpretation in epidemiological studies. Ann Epidemiol. 2019;29:1‐7. doi:10.1016/j.annepidem.2018.09.007 30342887 PMC6628690

[61] Bailey ZD , Feldman JM , Bassett MT . How structural racism works—racist policies as a root cause of U.S. racial health inequities. N Engl J Med. 2021;384:768‐773. doi:10.1056/NEJMms2025396 33326717 PMC11393777

[62] Geronimus AT . The weathering hypothesis and the health of African‐American women and infants: evidence and speculations. Ethn Dis. 1992;2:207‐221. 1467758

[63] Agbonlahor O , DeJarnett N , Hart JL , Bhatnagar A , McLeish AC , Walker KL . Racial/ethnic discrimination and cardiometabolic diseases: a systematic review. J Racial Ethn Health Disparities. 2023:1‐25. doi:10.1007/s40615-023-01561-1 PMC10044132 36976513

[64] Cuffee YL , Hargraves JL , Allison J . Exploring the association between reported discrimination and hypertension among African Americans: a systematic review. Ethn Dis. 2012;22:422‐431. 23140072

[65] Maier‐Hein KH , Neher PF , Houde J‐C , et al. The challenge of mapping the human connectome based on diffusion tractography. Nat Commun. 2017;8:1349. doi:10.1038/s41467-017-01285-x 29116093 PMC5677006

