# Supplementary material for: Unhealthy white matter connectivity, cognition, and racialization in older adults
Source: Alzheimers Dement. 2023 Oct 12;20(3):1483–96. doi: 10.1002/alz.13494 (PMC10947965; doi:10.1002/alz.13494)
Supplement: Supplementary file 1 — Supporting information [file ALZ-20-1483-s001.docx]

*Manuscript:* Unhealthy white matter connectivity, cognition, and racialization in older adults

**Supplementary Figure 1 Atlas used for lobar WMH volume derivation.** International Consortium for Brain Mapping (ICBM) 2009a Nonlinear Symmetric lobar atlas.

**
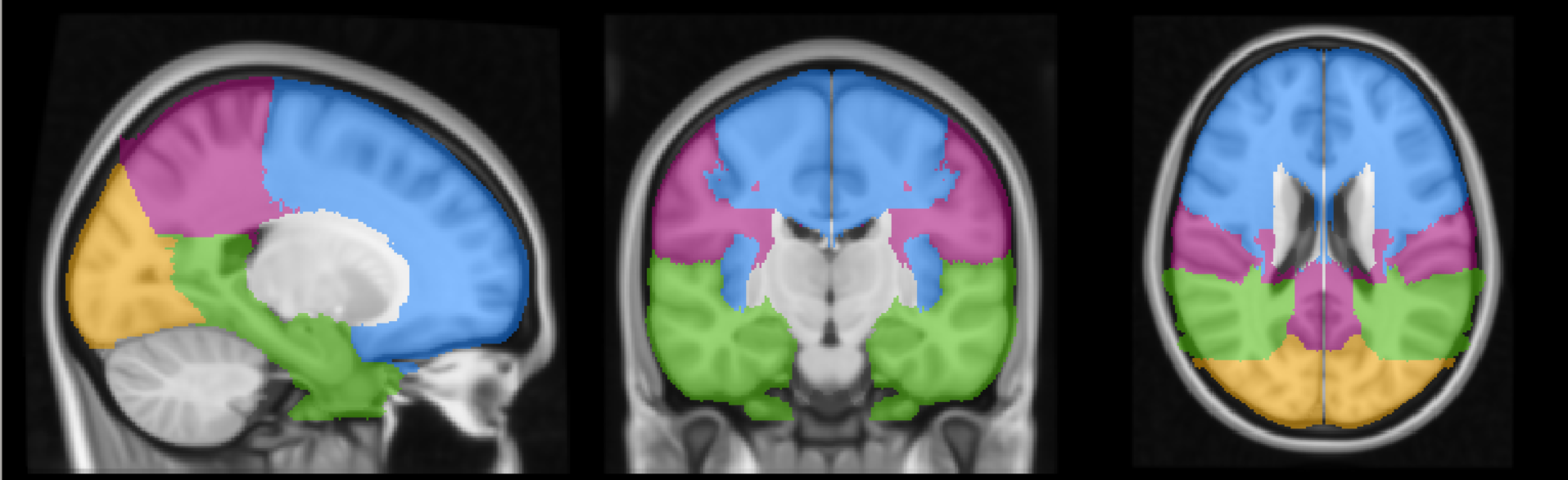
**

**Supplementary Table 1 FreeSurfer and CIC atlas regions used to derive UWMC for Aβ-affected regions**

| **Region name** |
| --- |
| ctx-lh-rostralanteriorcingulate |
| ctx-lh-caudalanteriorcingulate |
| ctx-rh-rostralanteriorcingulate |
| ctx-rh-caudalanteriorcingulate |
| ctx-lh-rostralmiddlefrontal |
| ctx-lh-superiorfrontal |
| ctx-lh-parstriangularis |
| ctx-lh-frontalpole |
| ctx-lh-parsopercularis |
| ctx-lh-caudalmiddlefrontal |
| ctx-lh-parsorbitalis |
| ctx-rh-rostralmiddlefrontal |
| ctx-rh-superiorfrontal |
| ctx-rh-parstriangularis |
| ctx-rh-frontalpole |
| ctx-rh-parsopercularis |
| ctx-rh-caudalmiddlefrontal |
| ctx-rh-parsorbitalis |
| ctx-lh-lateralorbitofrontal |
| ctx-lh-medialorbitofrontal |
| ctx-rh-lateralorbitofrontal |
| ctx-rh-medialorbitofrontal |
| ctx-lh-insula |
| ctx-rh-insula |
| ctx-lh-superiortemporal |
| ctx-lh-bankssts |
| ctx-rh-superiortemporal |
| ctx-rh-bankssts |
| ctx-lh-inferiorparietal |
| ctx-lh-superiorparietal |
| ctx-lh-supramarginal |
| ctx-rh-inferiorparietal |
| ctx-rh-superiorparietal |
| ctx-rh-supramarginal |
| ctx-lh-posteriorcingulate |
| ctx-lh-isthmuscingulate |
| ctx-rh-posteriorcingulate |
| ctx-rh-isthmuscingulate |
| ctx-lh-precuneus |
| ctx-rh-precuneus |
| Left-ventral striatum* |
| Right-ventral striatum* |

*CIC atlas

**Supplementary Table 2 Names of FreeSurfer regions used to derive UWMC for tau-affected regions**

| **Region name** |
| --- |
| ctx-lh-entorhinal |
| ctx-rh-entorhinal |
| ctx-lh-parahippocampal |
| ctx-rh-parahippocampal |
| ctx-lh-fusiform |
| ctx-rh-fusiform |
| ctx-lh-inferiortemporal |
| ctx-rh-inferiortemporal |
| ctx-lh-middletemporal |
| ctx-rh- middletemporal |
| Left-Amygdala |
| Right-Amygdala |

**Supplementary Table 3.** Characteristics of CoBRA participants at time of neuroimaging visit

|  | **Full cohort**  **n = 201** | **African American**  **n = 100** | **Non-Hispanic white**  **n = 101** | ***P*** |
| --- | --- | --- | --- | --- |
| **Demographics** |  |  |  |  |
| Age* (years) | 62 (57, 70) | 60 (56, 66) | 66 (59, 73) | 0.001^¶^ |
| Female^†^ | 134 (66.7%) | 71 (71.0%) | 63 (62.4%) | 0.19 |
| Education* (years) | 14 (12, 16) | 14 (12, 16) | 14 (12, 18) | 0.86 |
| Hours of physical activity per week* | 9.25 (3.75, 18.0) | 8.88 (3.63, 15.63) | 11.3 (7.00, 18.3) | 0.01^¶^ |
| **Cardiovascular risks** |  |  |  |  |
| Hypertension^†^ | 77 (38.3%) | 47 (47.0%) | 30 (29.7%) | 0.01^¶^ |
| Diabetes^†^ | 34 (16.9%) | 24 (24.0%) | 10 (9.90%) | 0.007^¶^ |
| High cholesterol^†^ | 71 (35.3%) | 34 (34.0%) | 37 (36.6%) | 0.69 |
| **Cognition** |  |  |  |  |
| MoCA score* | 25 (23, 27) | 24 (22, 26) | 27 (24, 28) | <0.0001^¶^ |
| Diagnosis^†^ |  |  |  |  |
| Alzheimer’s disease | 4 (1.99%) | 2 (2.00%) | 2 (1.98%) | 0.78 |
| MCI | 50 (24.9%) | 27 (27.0%) | 23 (22.8%) |  |
| Cognitively unimpaired | 147 (73.1%) | 71 (71.0%) | 76 (75.3%) |  |
| **AD-specific risks** |  |  |  |  |
| [^11^C]PiB SUVR*^,‡^ | 1.11 (1.06, 1.19) | 1.07 (1.04, 1.14) | 1.15 (1.08, 1.46) | <0.0001^¶^ |
| [^11^C]PiB positive^†,‡^ | 29 (16.2%) | 5 (5.32%) | 24 (28.2%) | <0.0001^¶^ |
| *APOE*4*^†^ | 63 (31.3%) | 35 (35.0%) | 28 (27.7%) | 0.26 |
| **Composite UWMC (unitless)** |  |  |  |  |
| Frontal lobe* | 0.14 (0.09, 0.26) | 0.16 (0.10, 0.30) | 0.12 (0.08, 0.22) | 0.03^¶^ |
| Parietal lobe* | 0.06 (0.01, 0.14) | 0.06 (0.01, 0.14) | 0.05 (0.01, 0.14) | 0.82 |
| Temporal lobe* | 0.18 (0.10, 0.26) | 0.21 (0.13, 0.28) | 0.15 (0.08, 0.23) | 0.001^¶^ |
| Occipital lobe* | 0.06 (0.01, 0.21) | 0.05 (0.01, 0.16) | 0.08 (0.08, 0.23) | 0.03^¶^ |
| Global* | 0.19 (0.13, 0.28) | 0.21 (0.15, 0.29) | 0.17 (0.12, 0.24) | 0.03^¶^ |
| High global UWMC* | 100 (49.6%) | 56 (56.0%) | 44 (43.6%) | 0.08 |
| **Composite WMH volume**^§^ **(unitless)** |  |  |  |  |
| Frontal lobe* | 55.3 (35.4, 111.1) | 68.9 (40.7, 127.6) | 49.2 (33.0, 91.5) | 0.002^¶^ |
| Parietal lobe* | 7.89 (1.24, 30.5) | 8.67 (1.3, 32.9) | 7.53 (1.17, 30.4) | 0.75 |
| Temporal lobe* | 29.5 (15.9, 48.9) | 34.8 (23.4, 57.1) | 23.3 (11.0, 41.6) | 0.0007^¶^ |
| Occipital lobe* | 2.77 (0.22, 13.6) | 1.25 (0.17, 8.78) | 4.86 (0.42, 18.4) | 0.01^¶^ |

*Median (25th percentile, 75th percentile)

^†^N (%)

^‡^*n* = 179

^§^Each lobar outcome (mm^3^) is normalized to intracranial volume (mm^3^), yielding unitless results

^¶^*P* < 0.05 between racialized groups

****MoCA = Montreal Cognitive Assessment; MCI = Mild Cognitive Impairment; PiB = Pittsburgh Compound-B; SUVR = standardized uptake value ratio; APOE = apolipoprotein; UWMC = unhealthy white matter connectivity; WMH = white matter hyperintensity
